# Differential importance of endothelial and hematopoietic cell GLP-1Rs for cardiometabolic versus hepatic actions of semaglutide

**DOI:** 10.1172/jci.insight.153732

**Published:** 2021-11-22

**Authors:** Brent A. McLean, Chi Kin Wong, Kiran Deep Kaur, Randy J. Seeley, Daniel J. Drucker

**Affiliations:** 1 Department of Medicine, Lunenfeld-Tanenbaum Research Institute, Mount Sinai Hospital, Toronto, Ontario, Canada.; 2 Department of Surgery, University of Michigan, Ann Arbor, Michigan.

**Keywords:** Endocrinology, Diabetes, Peptides

## Abstract

Glucagon-like peptide-1 receptor agonists (GLP-1RAs) are used to treat diabetes and obesity and reduce rates of major cardiovascular events, such as stroke and myocardial infarction. Nevertheless, the identity of GLP-1R–expressing cell types mediating the cardiovascular benefits of GLP-1RA remains incompletely characterized. Herein, we investigated the importance of murine *Glp1r* expression within endothelial and hematopoietic cells. Mice with targeted inactivation of *Glp1r* in Tie2^+^ cells exhibited reduced levels of *Glp1r* mRNA transcripts in aorta, liver, spleen, blood, and gut. *Glp1r* expression in bone marrow cells was very low and not further reduced in *Glp1r*^Tie2–/–^ mice. The GLP-1RA semaglutide reduced the development of atherosclerosis induced by viral PCSK9 expression in both *Glp1r*^Tie2+/+^ and *Glp1r*^Tie2–/–^ mice. Hepatic *Glp1r* mRNA transcripts were reduced in *Glp1r*^Tie2–/–^ mice, and liver *Glp1r* expression was localized to **γδ** T cells. Moreover, semaglutide reduced hepatic *Tnf*, *Abcg1*, *Tgfb1*, *Cd3g*, *Ccl2*, and *Il2* expression; triglyceride content; and collagen accumulation in high-fat, high-cholesterol diet–fed *Glp1r*^Tie2+/+^ mice but not *Glp1r*^Tie2–/–^ mice. Collectively, these findings demonstrate that Tie2^+^ endothelial or hematopoietic cell GLP-1Rs are dispensable for the antiatherogenic actions of GLP-1RA, whereas Tie2-targeted GLP-1R^+^ cells are required for a subset of the antiinflammatory actions of semaglutide in the liver.

## Graphical abstract

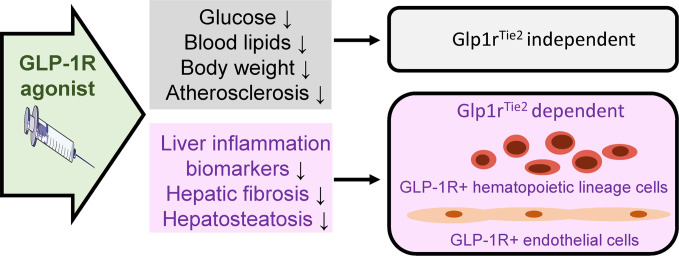

## Introduction

Glucagon-like peptide-1 (GLP-1) is a gut-derived incretin hormone secreted at low levels in the interprandial state, with circulating levels of GLP-1 rising briskly within minutes of meal ingestion (1, 2). The original description of GLP-1 action focused on its role as an incretin hormone that potentiated glucose-dependent insulin secretion (3, 4); these findings were subsequently extended to encompass the inhibition of glucagon secretion (5). GLP-1 also inhibits gastric emptying (6) and reduces appetite and food intake (7), leading to weight loss with chronic administration. Collectively, these mechanisms of action supported the clinical development of degradation-resistant GLP-1 receptor agonists (GLP-1RAs) for the treatment of type 2 diabetes (T2D) and obesity (1, 2).

The actions of GLP-1 are mediated by a single GPCR, a member of the class B GPCR family (8). The GLP-1R is widely expressed in several organs and cell types not classically linked to control of glucose homeostasis, including immune cells, endothelial and vascular smooth muscle cells, Brunner’s glands, and a subpopulation of cardiomyocytes (9). Notably, studies of GLP-1 action in animals and humans have demonstrated that GLP-1 decreases renal inflammation and albuminuria, reduces the extent of myocardial injury, attenuates the severity of experimental stroke, lowers blood pressure and postprandial lipemia, and exhibits antiatherogenic activity in sensitized mouse models prone to the development of atherosclerosis (10). These actions do not appear to be secondary to reduction of glycemia, as they have been detected in animals with normal glucose control.

Analysis of the time course of actions of glucose-lowering agents in outcome studies reveals that, unlike the rapid cardioprotective effects detected with use of sodium-glucose cotransporter protein-2 (SGLT-2) inhibitors (11), the cardiovascular benefits of GLP-1RAs take longer to emerge (10, 12), suggesting distinct mechanisms for these 2 classes of agents. Whereas SGLT-2 inhibitors are postulated to act in part through hemodynamic mechanisms, GLP-1RAs have been hypothesized to exert their cardiovascular benefits in part by attenuating the development of atherosclerosis. Indeed, studies of *Apoe*^–/–^ and *Ldlr*^–/–^ mice demonstrate that GLP-1RAs reduce the development of aortic root atherosclerosis, a finding associated with evidence for reduced tissue and systemic inflammation (13, 14). Nevertheless, the precise GLP-1R^+^ cell types transducing signals leading to the reduction of atherosclerosis remain unclear.

Here, we hypothesized that the actions of GLP-1RA to attenuate the extent of atherosclerosis require functional GLP-1Rs on endothelial cells (ECs) and/or hematopoietic lineage (HL) cells. Therefore, we crossed *Glp1r*^fl/fl^ mice with mice expressing Cre recombinase under the control of the *Tek* receptor tyrosine kinase (Tie2) promoter to generate *Glp1r*^Tie2–/–^ mice. We observed that loss of GLP-1Rs within the Tie2^+^ expression domain does not modify the development of experimental atherosclerosis or the antiatherogenic activity of the GLP-1RA semaglutide. Unexpectedly, we detected reduction of hepatic *Glp1r* expression in *Glp1r*^Tie2–/–^ mice. Analysis of purified cell populations identified *Glp1r* expression by qPCR and by RT-PCR using primers that span the entire open reading frame in a subset of intrahepatic γδ T cells. Semaglutide reduced hepatic cytokine expression in *Glp1r*^Tie2+/+^mice; however, these actions were blunted in *Glp1r*^Tie2–/–^ mice. Moreover, the GLP-1RA exendin-4 (Ex-4) directly reduced *Ifng* mRNA transcripts in nonhepatocyte (NH) liver cells isolated from *Glp1r*^Tie2+/+^ mice but not from cells isolated from *Glp1r*^Tie2–/–^ mice. Collectively, these findings demonstrate that the antiatherogenic actions of GLP-1RAs do not require GLP-1Rs within ECs or HL cells; however, reduction of *Glp1r* expression within cells targeted by Tie2-Cre attenuates semaglutide-mediated reduction of cytokine expression, triglyceride accumulation, and fibrosis in the high-fat, high-cholesterol (HFHC) diet–fed mouse liver.

## Results

### GLP-1R agonism with liraglutide reduces atherosclerosis in Ldlr^–/–^ mice.

Our previous studies of atherosclerosis failed to demonstrate a clear reduction in aortic plaque burden in *Apoe*^–/–^ mice treated with the long-acting GLP-1RA taspoglutide (15). Accordingly, prior to undertaking more detailed studies of the GLP-1R and atherosclerosis using mouse genetics, we first investigated the effects of daily administration of liraglutide, a GLP-1RA shown to (a) reduce experimental atherosclerosis in mice (16, 17) and (b) decrease major adverse cardiovascular events in humans (12). Liraglutide administration (200 μg/kg/d) for 18 weeks reduced aortic plaque area in HFHC diet–fed LDL receptor (*Ldlr^–/–^*) mice (Supplemental Figure 1A; supplemental material available online with this article; https://doi.org/10.1172/jci.insight.153732DS1). Notably, liraglutide-treated mice also exhibited reduced body weight, fat mass, lean mass, and liver weights (Supplemental Figure 1, B–D).

### Glp1r mRNA transcripts are enriched within Tie2^+^ aortic ECs.

**Figure 1:**
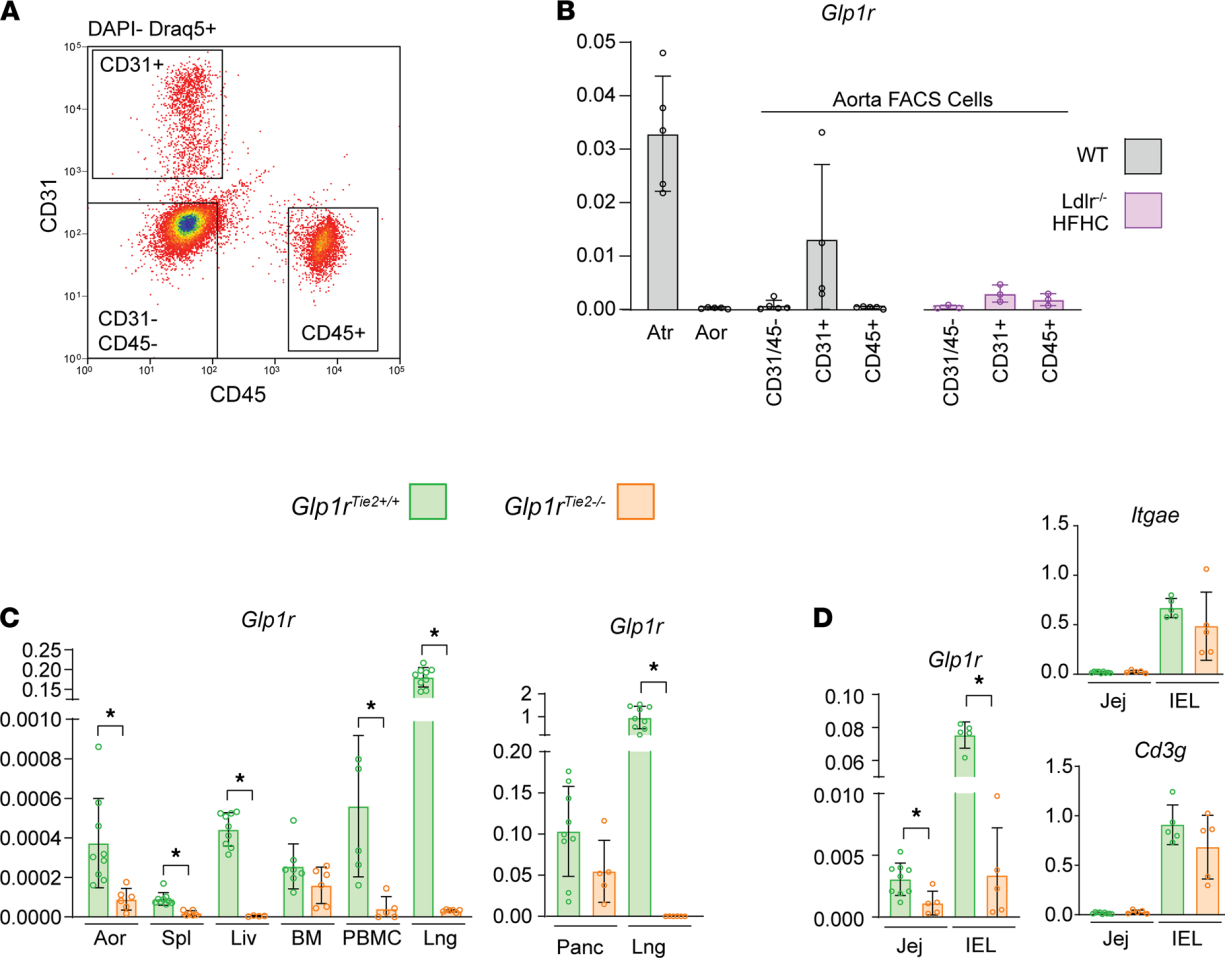
Aortic endothelial cells are enriched for Glp1r expression, and Tie2-directed recombination reduces Glp1r mRNA in multiple organs. Isolated cells from mouse aortas were subjected to FACS cytometry collection. (**A**) Gating strategy. (**B**) *Glp1r* versus *Ppia* expression levels in whole heart atria (Atr) and whole aorta (Aor) as well as FACS-collected cells that are endothelial cells (CD31^+^), immune cells (CD45^+^), or remaining cell types (CD31^–^/CD45^–^) from either chow-fed WT mice or *Ldlr^–/–^* mice fed HFHC diet for 18 weeks. For whole tissue, *n =* 5. FACS-derived cells were isolated from aortas pooled from 5 to 9 mice to generate *n =* 3–5 independent pooled samples for analysis. (**C**) *Glp1r* expression was analyzed in tissues from *Glp1r*^Tie2+/+^ and *Glp1r*^Tie2–/–^ mice and is depicted relative to *Ppia* mRNA transcripts in whole aorta, spleen (Spl), liver (Liv), bone marrow, PBMCs, and lung (Lng) (left). For pancreas and lung, relative *Glp1r* mRNA was normalized to levels of *Rpl32* (right) (*n =* 6–9). (**D**) Gut sections from the jejunum (Jej) and samples enriched for gut IELs were assessed for *Glp1r* versus *Ppia* expression and IEL markers *Itgae* and *Cd3g* to confirm successful IEL purification (*n =* 5–8). Data are presented as mean ± SD, with individual data points shown. *P < 0.05, Student’s *t* test for effect of *Glp1r*^Tie2+/+^ versus *Glp1r*^Tie2–/–^ genotype.

Considering roles for GLP-1R^+^ cells in atherosclerosis, GLP-1R expression has been described within ECs (18) and vascular smooth muscle cells (19, 20), and within immune cell populations (21), with the highest levels of immune cell GLP-1R expression detected within intestinal intraepithelial lymphocytes (IELs) (22). Notably, the antiatherogenic activity of GLP-1RA has been linked to direct reduction of vascular smooth muscle cell proliferation (16). Nevertheless, precise identification of vascular GLP-1R^+^ cell types linked to the reduction of atherosclerosis following treatment with GLP-1RA remains uncertain (9). We used enzymatic and mechanical digestion of mouse aortas, followed by FACS cytometry to collect major cell types for mRNA analysis. Expression of the *Glp1r* was enriched in CD31^+^ ECs isolated from both healthy aortas as well as from atherosclerotic aortas from *Ldlr*^–/–^ mice fed a HFHC diet (Figure 1, A and B). Interestingly, levels of *Glp1r* mRNA transcripts were reduced in CD31^+^ cells isolated from HFHC diet–fed *Ldlr*^–/–^ mouse aortas (Figure 1B). Accordingly, to target the *Glp1r* within the CD31^+^ EC population, we crossed *Glp1r*^fl/fl^ mice with mice expressing Cre recombinase under the *Tie2* (EC-specific receptor tyrosine kinase [TEK]) promoter to generate *Glp1r*^Tie2–/–^ mice. Analysis of tissues from *Glp1r*^Tie2–/–^ mice revealed knockdown of *Glp1r* in the aorta, spleen, liver, PBMCs, and lung but not in bone marrow (Figure 1C). Notably, pancreatic *Glp1r* expression was unchanged, whereas *Glp1r* expression was markedly reduced in lung tissue from *Glp1r*^Tie2–/–^ mice (Figure 1C). Consistent with expression of Tie2-Cre within HL cells (23), we also observed reduction of *Glp1r* mRNA transcripts in jejunum and in purified small bowel IELs, the major GLP-1R^+^ cell type within the small bowel. Analysis of *Itgae* and *Cd3g* mRNA was used to demonstrate successful enrichment of gut IELs (Figure 1D).

### GLP-1RAs attenuate atherosclerosis independent of the endothelial and hematopoietic Glp1r.

**Figure 2:**
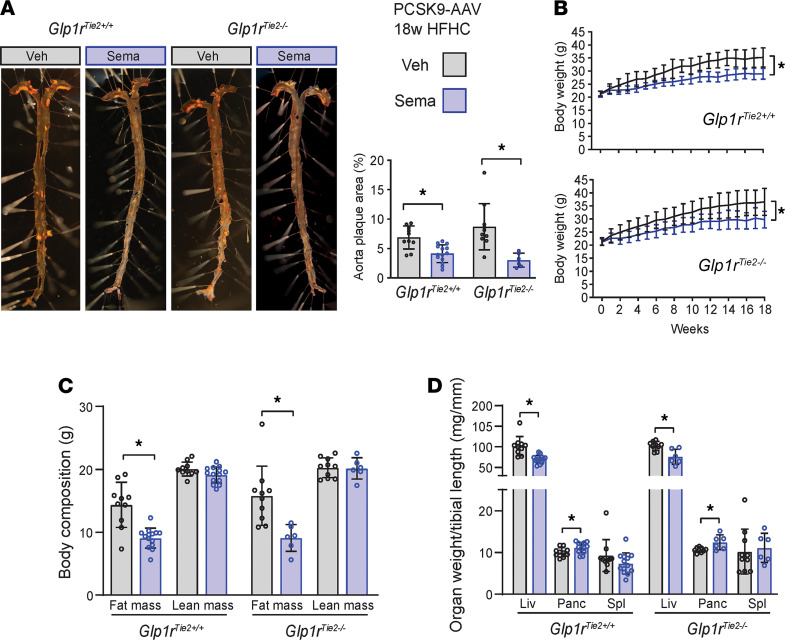
Aortic atherosclerosis is attenuated by semaglutide treatment in *Glp1r*^Tie2+/+^ and *Glp1r*^Tie2–/–^ mice. *Glp1r*^Tie2+/+^ and *Glp1r*^Tie2–/–^ mice were given a PCSK9-AAV injection, followed by HFHC-diet feeding and daily administration of either semaglutide (Sema; 10 μg/kg) or an equal volume PBS vehicle (veh) for 18 weeks (PCSK9+HFHC diet protocol). (**A**) Whole en face mounted aortas stained with Sudan IV for atherosclerotic plaques; representative images and quantification. (**B**) Weekly body weight over the treatment period. (**C**) Body composition measured 1 week before the end of experiment. (**D**) Liver (Liv), pancreas (Panc), and spleen (Spl) weights are shown relative to tibial length (*n =* 6–14). Data are presented as mean ± SD, with individual data points shown. *P < 0.05 for Sema effect, 2-way ANOVA multiple-comparison test.

We next assessed whether semaglutide, a GLP-1RA approved for the treatment of T2D and obesity, would reduce the development of atherosclerosis to a similar extent in *Glp1r*^Tie2+/+^ versus *Glp1r*^Tie2–/–^ mice. To promote the development of atherosclerosis, HFHC diet–fed mice were administered proprotein convertase subtilisin/kexin type 9–adeno-associated virus (PCSK9-AAV) followed by daily administration of semaglutide or saline (control) for 18 weeks. Semaglutide-treated mice exhibited reduced aortic atherosclerotic plaque area measured by whole aorta en face staining (Figure 2A). Notably, in the absence of semaglutide, the extent of atherosclerosis was not different in *Glp1r*^Tie2–/–^ versus *Glp1r*^Tie2+/+^ mice (Figure 2A). Body weight was reduced in both *Glp1r*^Tie2–/–^ and *Glp1r*^Tie2+/+^ mice after 18 weeks of daily semaglutide administration (Figure 2B). Semaglutide therapy reduced fat but not lean mass (Figure 2C) and decreased liver weight but not spleen weight, irrespective of genotype (Figure 2D). Consistent with previous findings (24, 25), pancreatic weight was increased following semaglutide administration (Figure 2D).

**Figure 3:**
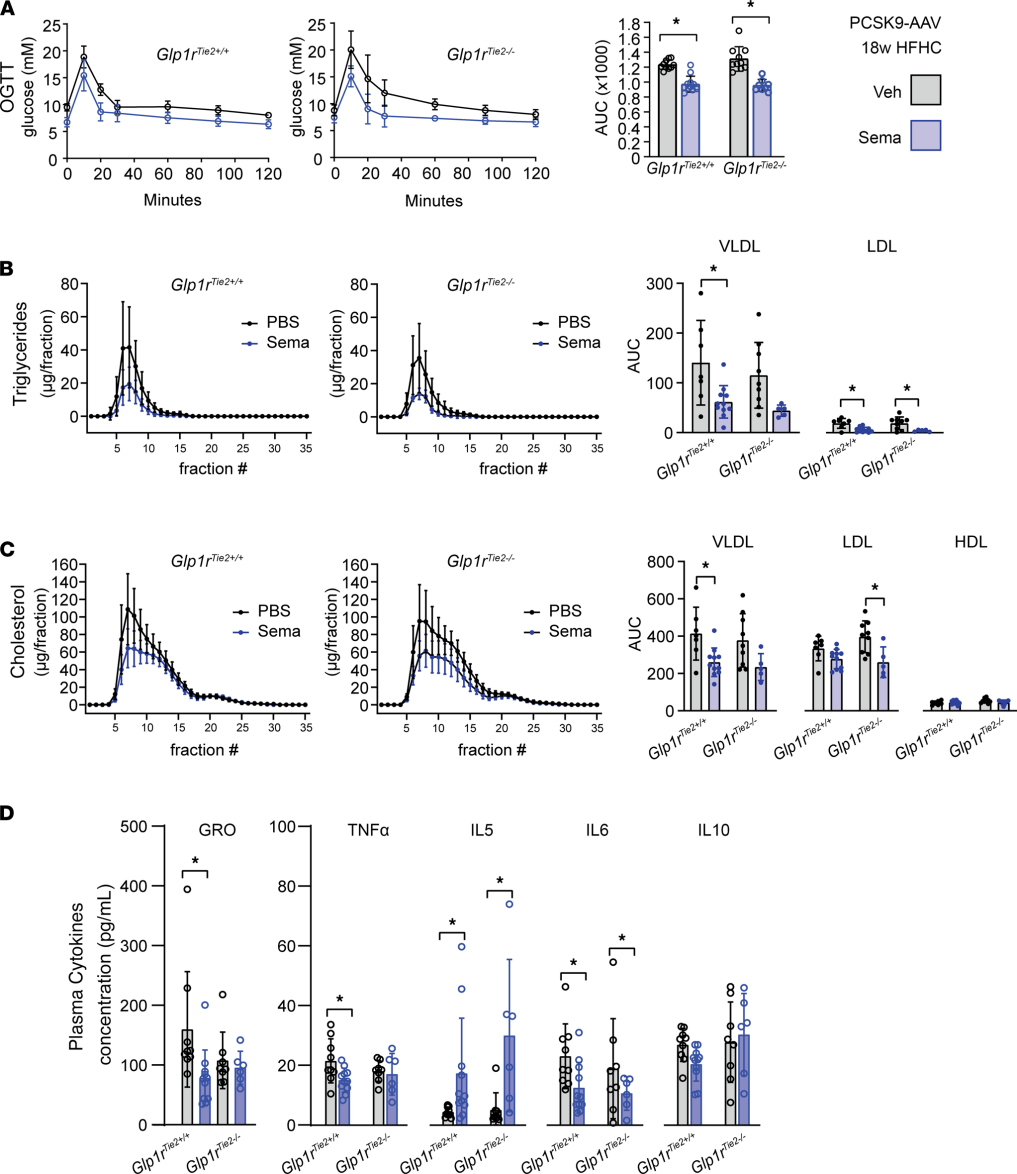
Semaglutide attenuates glycemic excursion and levels of circulating cholesterol, triglycerides, and cytokines in *Glp1r*^Tie2+/+^ and *Glp1r*^Tie2–/–^ mice. PCSK9-treated HFHC diet–fed mice were tested for blood glucose at weeks 12–13 of the 18-week PCSK9+HFHC diet protocol, with daily vehicle (Veh) or semaglutide (Sema; 10 μg/kg) administration. Mice were fasted for 5 hours followed by administration of 1.5 g/kg glucose for an oral glucose tolerance test (OGTT). (**A**) The area under the curve (AUC) was calculated to compare Veh with Sema treatment in *Glp1r*^Tie2+/+^ and *Glp1r*^Tie2–/–^ mice. At weeks 16–17 of the treatment protocol, mice were fasted for 5 hours and blood was collected for fast protein lipid chromatography: (**B**) triglyceride and (**C**) cholesterol levels in VLDL, LDL,and HDL fractions were measured. (**D**) Cytokines were measured in plasma obtained from terminal bleeds at the end of the experiment (*n =* 6–14). Data are presented as mean ± SD, with individual data points shown. *P < 0.05 for Sema effect, 2-way ANOVA multiple-comparison test.

Metabolic studies revealed that semaglutide reduced glycemic excursion after oral glucose administration in both *Glp1r*^Tie2+/+^ and *Glp1r*^Tie2–/–^ mice (Figure 3A). Blood lipid analysis revealed that semaglutide reduced both triglyceride and cholesterol levels; reduction of circulating lipoproteins following semaglutide was evident within both VLDL and LDL subfractions, with minor differences between genotypes (Figure 3, B and C). As GLP-1RAs reduce tissue and systemic inflammation in the context of experimental atherosclerosis (14, 26), we assessed circulating levels of plasma cytokines. Semaglutide treatment increased levels of IL-5 and decreased levels of IL-6 in both *Glp1r*^Tie2+/+^ and *Glp1r*^Tie2–/–^ mice (Figure 3D). Interestingly, semaglutide reduced plasma levels of KC/GRO (also known as CXCL1) and TNF-α in *Glp1r*^Tie2+/+^ mice but not in *Glp1r*^Tie2–/–^ mice, whereas no treatment or genotype effects were evident in analysis of circulating IL-10 (Figure 3D).

### Semaglutide does not promote regression of established atherosclerosis in PCSK9-AAV–treated HFHC diet–fed mice.

To determine whether semaglutide administration is capable of promoting regression of already established atherosclerosis, WT mice were injected with PCSK9-AAV, maintained for 16 weeks on a HFHC diet, and then switched to regular chow for a 1-week washout period. Groups of mice were then randomized to receive either daily semaglutide (10 μg/kg/d) or an equal volume of once daily saline as a vehicle control for 6 weeks (Supplemental Figure 2A). Whole aorta en face imaging showed that neither vehicle-treated nor semaglutide-treated groups exhibited reduced aorta plaque area compared with the baseline controls (Supplemental Figure 2B), even though semaglutide administration caused body weight loss but did not reduce liver or spleen weight (Supplemental Figure 2, C and D).

### Semaglutide reduces markers of liver injury and inflammation in PCSK9-AAV–treated HFHC-fed mice.

**Figure 4:**
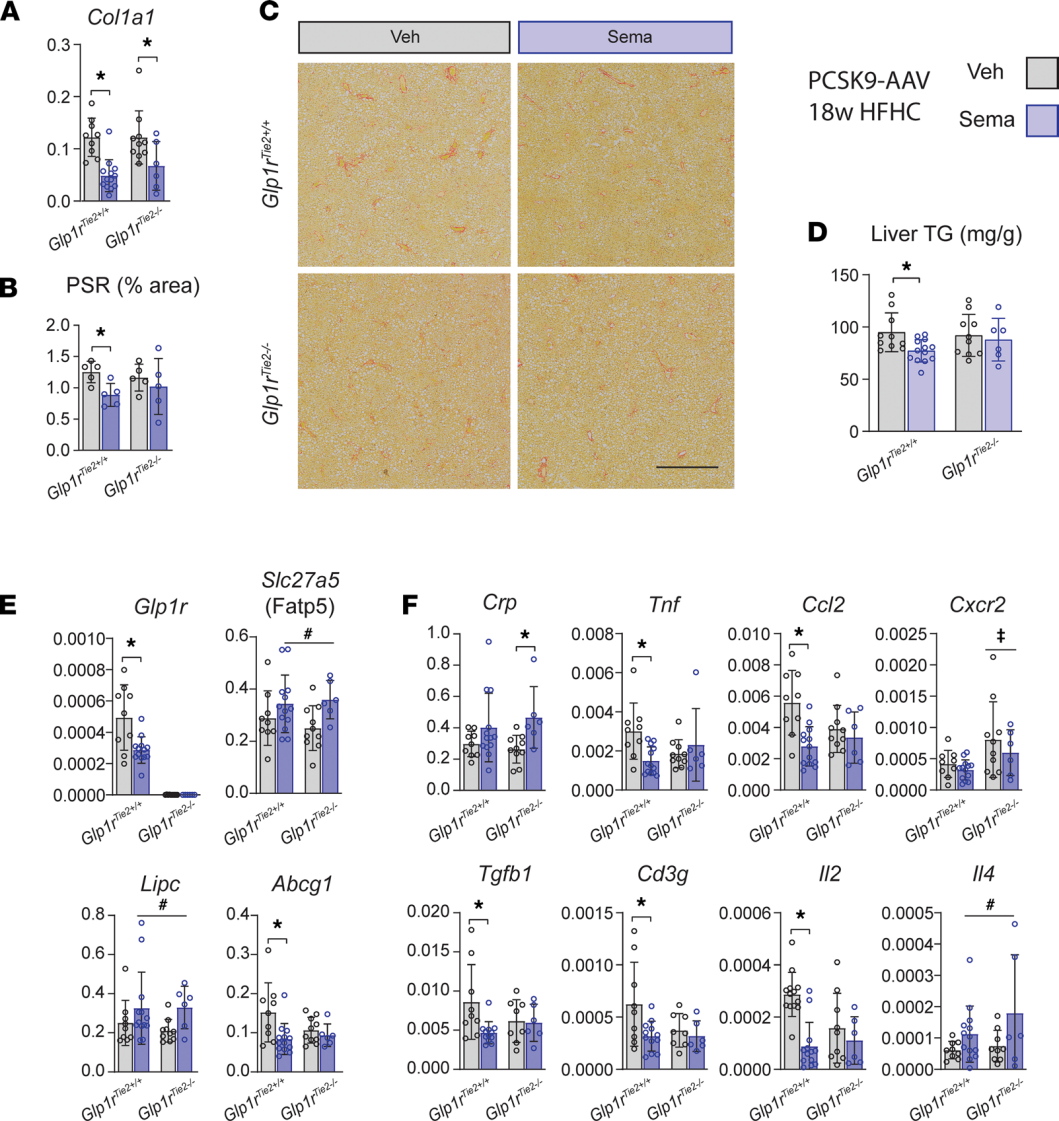
Semaglutide reduces liver fibrosis and hepatic cytokine gene expression in *Glp1r*^Tie2+/+^ mice but not in *Glp1r*^Tie2–/–^ mice. Analysis of liver tissue from PCSK9+HFHC-fed mice treated with vehicle (Veh) or semaglutide (Sema) for 18 weeks: (**A**) *Col1a1* versus *Ppia* expression, (**B**) Picrosirius red^+^ (PSR^+^) collagen staining quantification (*n =* 5), and (**C**) representative images (scale bar: 500 μm). (**D**) Liver tissue triglycerides (TG), (**E**) liver expression of *Glp1r* and metabolic regulators, and (**F**) inflammation markers versus *Ppia* (*n =* 6–14). Data are presented as mean ± SD, with individual data points shown. *P < 0.05 for Sema effect, 2-way ANOVA multiple-comparison test; ^#^*P <* 0.05, 2-way ANOVA effect for treatment; ‡*P <* 0.05, 2-way ANOVA effect for genotype.

Detection of reduced *Glp1r* expression in the livers of *Glp1r*^Tie2–/–^ mice (Figure 1C) prompted us to assess whether hepatic indices of metabolism or inflammation were differentially regulated in atherosclerosis-prone, PCSK9-AAV–treated, HFHC diet–fed mice treated with or without semaglutide. Semaglutide treatment reduced hepatic *Col1a1* expression in both *Glp1r*^Tie2+/+^ and *Glp1r*^Tie2–/–^ mice (Figure 4A). The extent of Picrosirius red^+^ collagen staining was reduced in semaglutide-treated *Glp1r*^Tie2+/+^ mice but not in *Glp1r*^Tie2–/–^ mice (Figure 4, B and C). Semaglutide reduced liver triglyceride levels in *Glp1r*^Tie2+/+^ mice but not in *Glp1r*^Tie2–/–^ mice (Figure 4D). Interestingly, chronic daily treatment with semaglutide for 18 weeks reduced *Glp1r* expression in the liver of *Glp1r*^Tie2+/+^mice (Figure 4E). Levels of mRNAs for liver-specific fatty acid transport protein 5 (Fatp5) encoded by *Slc27a5* and hepatic lipase (*Lipc*) were increased by semaglutide treatment independent of genotype, whereas hepatic *Abcg1* mRNA transcripts were decreased in livers from *Glp1r*^Tie2+/+^ mice but not *Glp1r*^Tie2–/–^ mice (Figure 4E). Further genotype-dependent differences were evident upon analysis of hepatic biomarkers of inflammation. Semaglutide reduced levels of *Tnf*, *Ccl2*, *Tgfb1*, *Cd3g*, and *Il2* mRNA transcripts in livers from *Glp1r*^Tie2+/+^ mice but not *Glp1r*^Tie2–/–^ mice (Figure 4F), whereas levels of hepatic *Crp* mRNA transcripts were higher in semaglutide-treated *Glp1r*^Tie2–/–^ mice. Two-way ANOVA analysis indicated that semaglutide increased levels of hepatic *Il4* mRNA, whereas *Cxcr2* mRNA transcripts were higher in the liver from *Glp1r*^Tie2–/–^ mice (Figure 4F).

### Loss of the endothelial and hematopoietic Glp1r does not affect levels of circulating endogenous GLP-1.

Recent studies of integrin β7^–/–^ mice revealed that loss of the IEL population, including GLP-1R^+^ IELs, was associated with increased circulating levels of GLP-1 (27). As *Glp1r*^Tie2–/–^ mice exhibited marked reduction in IEL *Glp1r* expression (Figure 1D), we assessed whether more selective reduction of IEL GLP-1R expression, as opposed to loss of the entire IEL population and systemic consequences arising from loss of integrin β7, would upregulate plasma levels of GLP-1. Notably, basal levels of circulating GLP-1 were not different in HFHC-fed *Glp1r*^Tie2–/–^ mice, and oral administration of glucose or olive oil induced plasma levels of total GLP-1 to a similar extent in both *Glp1r*^Tie2+/+^ and *Glp1r*^Tie2–/–^ mice (Supplemental Figure 3). Hence, reduction of the IEL *Glp1r* is not sufficient to enhance basal or nutrient-stimulated L cell GLP-1 secretion in *Glp1r*^Tie2–/–^ mice.

### Liver Glp1r localizes to a subset of T cells.

The detection and cellular localization of *Glp1r* expression in the liver has been the subject of some controversy (9). Whereas several studies failed to detect *Glp1r* mRNA transcripts encoding a canonical functional GLP-1R in liver and isolated hepatocytes (28, 29), low level expression of *Glp1r* has been detected in RNA from mouse liver (9, 15), previously localized to neural fibers in the proximity of the hepatic portal vein (30, 31). To ascertain the identity of *Glp1r*^+^ cells in the liver, we performed FACS cytometry of cells obtained following liver perfusion and enzymatic digestion, isolating fractions for ECs (CD31^+^), Kupffer cells (CD45^+^AF^+^), non-Kupffer immune cells (CD45^+^AF^–^), and unstained cells (CD31^–^CD45^–^; Supplemental Figure 4A). Enrichment of *Glp1r* expression was detected in CD45^–^CD31^+^ ECs and CD45^+^AF^–^ non-Kupffer immune cells (Supplemental Figure 4B). *Glp1r* mRNA transcripts were not enriched in liver NK cells (Supplemental Figure 4, C and D) or NK T cells (Supplemental Figure 4, E and F). Analysis of *Rag2/Il2rg* double-knockout mice known to exhibit absence of functional T, B, and NK cells showed reduced hepatic *Glp1r* expression compared with WT controls (Supplemental Figure 4G).

**Figure 5:**
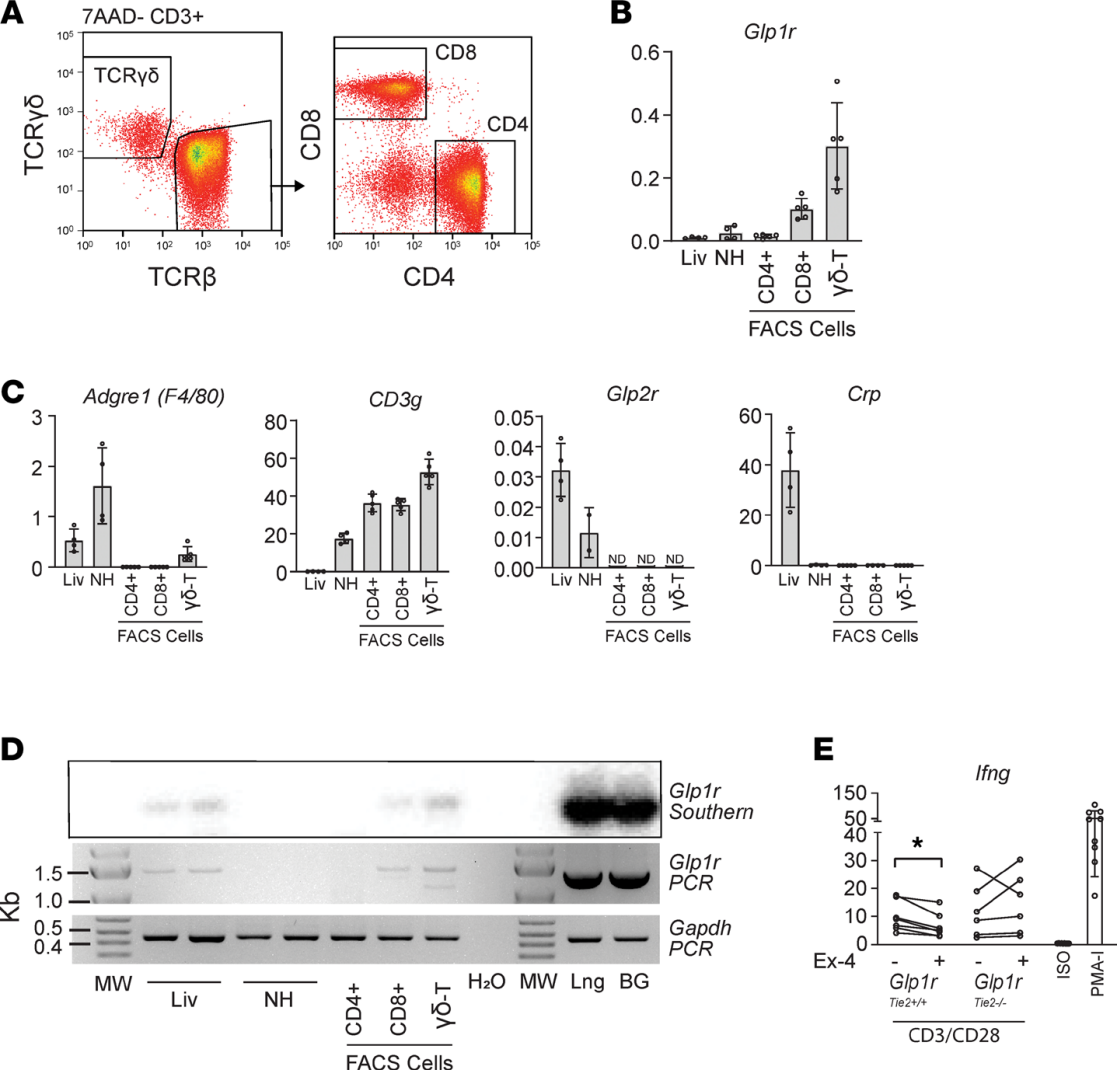
Glp1r expression in the liver is localized to CD8α and γδ T cells. (**A**) Cytometry gating strategy shown for liver T cell populations. (**B**) Whole liver (Liv), Percoll-purified nonhepatocyte cells (NH), and FACS-collected NH cells positive for CD3^+^TCRγδ or CD3^+^TCRβ (γδ-T) and either CD8α (CD8^+^) or CD4 (CD4^+^) were analyzed for expression of *Glp1r* as well as (**C**) *Adgre1*, *Cd3g*, *Glp2r*, and *Crp* as markers of macrophages, T cells, stellate cells, and hepatocytes, respectively, versus *Tbp* (*n =* 4–5). Data are presented as mean ± SD, with individual data points shown. (**D**) Full-length *Glp1r* transcript was amplified in liver as well as CD8^+^ and TCR γδ^+^ T cell populations; lung (Lng) and Brunner’s glands (BG) are positive controls (representative of 3 replicates). (**E**) IFN-γ (*Ifng*) expression in freshly isolated NH cells from *Glp1r*^Tie2+/+^ (*n =* 7) and *Glp1r*^Tie2–/–^ (*n =* 6) mice were cultured overnight with CD3/CD28 stimulation with or without exendin-4 (Ex-4; 50 nM), negative isotype control (ISO), and PMA-ionomycin (PMA-I) positive control (*n =* 9). *P < 0.05 paired *t* test.

Analysis of FACS-sorted CD3^+^ T cell subpopulations revealed that *Glp1r* mRNA transcripts were enriched within CD8^+^ and γδ T cells (Figure 5, A and B), with cellular identities confirmed through analysis of *Adgre1* (macrophages), *Cd3g* (T cells), *Glp2r* (hepatic stellate cells), and *Crp* (hepatocytes) expression (Figure 5C). We next utilized conventional PCR to amplify a transcript spanning the *Glp1r* mRNA open reading frame sequence. These experiments detected a *Glp1r* mRNA transcript capable of encoding a GLP-1R protein in liver and purified T cell populations but not in the NH fraction of WT mice (Figure 5D). To test the capacity of GLP-1R to functionally modulate T cell activation, we cultured NH preparations from *Glp1r*^Tie2+/+^ and *Glp1r*^Tie2–/–^ mice with overnight (20 hour) anti-CD3/28 stimulation and paired samples cultured with and without Ex-4 (50 nM); *Ifng* expression, indicative of T cell activation, was significantly reduced by Ex-4 in *Glp1r*^Tie2+/+^ cells but not in *Glp1r*^Tie2–/–^ cells (Figure 5E).

## Discussion

The development of atherosclerosis is a complex process involving contributions from vascular ECs and smooth muscle cells; HL immune cells, including macrophages, lipoprotein particles, local and systemic inflammatory mediators, and circulating blood cells; and a host of local and systemic factors that affect blood vessel health (32). GLP-1 is known to modify several risk factors for atherosclerosis, including systemic and vascular inflammation, blood pressure, and circulating levels of triglyceride-rich lipoproteins (10). We hypothesized that either ECs or HL cells could mediate important actions of GLP-1RAs in atherosclerosis and metabolic disease. ECs play multiple roles in the development of vascular pathology, enabling leukocyte infiltration of vascular lesions and paracrine regulation of vascular smooth muscle, as well as contributing to control of inflammation in atherosclerotic lesions (33). HL leukocytes, most prominently infiltrating monocytes and macrophages, are central mediators of atherosclerosis plaque progression; however, roles for platelets, eosinophils, T cells, and diverse immune cells have also been described (34). Accordingly, we utilized the Tie2 promoter to direct Cre expression within both ECs and HL cells (35). Our data indicate that CD45^+^ cells (representative of the majority of nonerythroid HL cells) isolated from the healthy or diseased mouse aorta, do not express appreciable levels of *Glp1r*. We used female mice for the majority of this study, with the exception of *Ldlr*^–/–^ mice (Supplemental Figure 1), which were male. We gave preference to female mice for these chronic studies because they are less likely to fight under conditions of chronic housing and daily injections, and female *Ldlr*^–/–^ mice were reported to have greater atherosclerosis burden compared with male mice (36). Notably, GLP-1RA therapy has not been shown to display sex-dependent effects in the control of glucose metabolism or cardioprotection.

Analysis of *Glp1r* expression in organs from *Glp1r*^Tie2–/–^ mice revealed reduced *Glp1r* expression in the aorta, spleen, liver, PBMCs, and lung. Knockdown of *Glp1r* mRNA in the *Glp1r*^Tie2–/–^ aorta is consistent with our detection of *Glp1r* expression within purified ECs isolated from the aorta. Similarly, marked reduction of *Glp1r* mRNA transcripts in the lungs of *Glp1r*^Tie2–/–^ mice is in agreement with previous detection of *Glp1r* expression within mouse lung ECs (18) and with independent reports of EC *Glp1r* expression in a mouse lung single-cell RNA-seq data set (37). The expression of *Glp1r* is extremely low and not reduced in *Glp1r*^Tie2–/–^ mouse bone marrow. In contrast, and consistent with previous reports (22, 27), *Glp1r* is readily detectable in small bowel IELs and markedly reduced in IELs isolated from *Glp1*r^Tie2–/–^ mice.

An unresolved question is the extent to which weight loss associated with GLP-1RA utilized in animal studies contributes to the attenuation of atherosclerosis. Here we used a dose of 10 μg/kg/d of semaglutide, demonstrating that reduction of aortic atherosclerosis does not require a functional GLP-1R within ECs or HL cells using the PCSK9-AAV+HFHC diet model of atherosclerosis, which closely mimics the phenotypes exhibited by *Ldlr*^–/–^ mice (38). Mice treated with semaglutide exhibited improved glucose tolerance, decreased blood lipids, and reduced body weight, consistent with the actions of GLP-1RAs in people with T2D. We chose this dose of semaglutide because it was in a range previously shown to cause atherosclerosis reduction in *Ldlr*^–/–^ mice over a similar treatment schedule (14). Considering that our primary aim was to determine if any effects of semaglutide treatment were lost with selective deletion of the GLP-1R, we chose to not attempt to further reduce the semaglutide dose to an extent that would eliminate body weight loss and risk obscuring the importance of *Glp1r*^Tie2+/+^ cells.

Several studies using GLP-1RAs to attenuate atherosclerosis in *Apoe*^–/–^ or *Ldlr*^–/–^ mice have also reported concomitant weight loss (14, 17, 39). However, a substantial number of studies demonstrate weight loss–independent antiatherogenic actions of GLP-1RA in mice (40, 41). For example, Rakipovski et al. used weight-matched controls, generated using treatment with a food intake–reducing agent, to infer that the antiatherogenic effects of liraglutide were independent of changes in body weight in *Apoe*^–/–^ mice (14). Furthermore, Bruen and colleagues demonstrated attenuation of atherosclerosis in liraglutide-treated *Apoe*^–/–^ mice without differences in body weight between groups (26).

Among the potentially new insights reported here is the localization of hepatic *Glp1r* expression to a subset of γδ T cells. *Glp1r* expression is extremely low or reported as undetectable in whole liver RNA, often below the threshold level of detection using RNA-Seq (9, 42). *Glp1r* transcriptional sequences directed low level expression of a fluorescent tdTomato reporter protein within murine hepatic ECs; however, whether endogenous *Glp1r* mRNA transcripts are also detected in these cells was not examined (43). Interestingly, we observed that hepatic *Glp1r* expression was reduced in semaglutide-treated HFHC diet–fed mice. Importantly, semaglutide also reduced the hepatic expression of biomarkers of inflammation and metabolic regulation, fibrosis, and hepatic triglyceride levels in HFHC diet–fed *Glp1r*^Tie2+/+^ mice but not in *Glp1r*^Tie2–/–^ mice, despite comparable weight loss in these 2 groups of mice. Hence, GLP-1R^+^ cells within the *Tie2* expression domain contribute to the antiinflammatory and antisteatotic actions of semaglutide in the mouse liver. Interestingly, vehicle-treated *Glp1r*^Tie2–/–^ mice mirrored *Glp1r*^Tie2+/+^ mice in most hepatic parameters assessed, with a trend of lower levels of some mRNA transcripts, including *Tnf*, *Ccl2*, *Tgfb1*, *Cd3g*, and *Il2*. Whether basal levels of GLP-1Rs within Tie2^+^ cells are important for regulation of hepatic cytokine expression is not clear and requires further study.

The current experimental design does not allow us to infer whether the antiinflammatory actions of semaglutide engage GLP-1Rs within intrahepatic γδ T cells, gut IELs, ECs, circulating PBMCs, or other Tie2^+^ cell populations. Ascertaining the putative importance of GLP-1R expression within γδ T cell subtypes is likely to be challenging, considering the low abundance of γδ T cells in most secondary lymphoid organs and the relatively low expression levels of *Glp1r* within these cells. γδ T cells may contribute to IL-17A or IFN-γ production in some settings depending on their lineage (44). We found that IL-17A levels were very low in blood plasma (20 samples below detection limit, 15 samples between 0.6 and 21.0 pg/ml). Similarly, hepatic *Il17a* mRNA transcripts were marginally detectable in liver (24 samples undetectable, 11 samples with CT values of 36–39) in our PCSK9-AAV+HFHC-treated mice. Hence, our present data are very limited in this regard, and we cannot rule out a possible role for GLP-1RA treatment in modulating IL-17A in this or other settings. Further studies will be required to characterize the subtype and identity of GLP-1R–expressing γδ T cells in mice and their similarities and differences to human γδ T cells.

Studies of integrin β7^–/–^ mice revealed protection from atherosclerosis, findings attributed to elevated levels of endogenous GLP-1 secondary to loss of the GLP-1R^+^ IEL population. Intriguingly, absence of GLP-1R^+^ IELs in these mice was associated with upregulation of enteroendocrine L cell number and enhanced GLP-1 synthesis and secretion (27). In contrast to phenotypes observed in integrin β7^–/–^ mice, *Glp1r*^Tie2–/–^ mice with marked reduction of IEL *Glp1r* expression did not exhibit any differences in circulating levels of GLP-1 or any baseline differences in atherosclerosis plaque burden. Moreover, integrin β7^–/–^ mice exhibited increased food intake, energy expenditure, and body temperature, whereas body weight differences were not detected in *Glp1r*^Tie2–/–^ mice (Figure 2B). Hence, our current data reveal that knockdown of *Glp1r* within IELs does not phenocopy key findings arising in integrin β7^–/–^ mice with complete elimination of the GLP-1R^+^ IEL population. Moreover, the actions of semaglutide to reduce body weight, improve glucose tolerance, and decrease circulating levels of triglyceride-enriched lipoproteins were not diminished in *Glp1r*^Tie2–/–^ mice, revealing that GLP-1Rs within the Tie2 expression domain, including IEL GLP-1R, are not required for these metabolic actions of semaglutide.

The majority of preclinical studies examining how GLP-1RAs modify atherosclerosis have employed treatment regimens focused on attenuation of the development of atherosclerosis in genetically sensitized mice. In contrast, Bruen and colleagues demonstrated regression of aortic atherosclerosis following 4 weeks of liraglutide administration in *Apoe*^–/–^ mice (26). We detected attenuation of atherosclerosis in HFHC diet–fed mice with PCSK9-AAV–induced atherosclerosis following 18 weeks of semaglutide treatment, yet we were not able to detect regression of aortic atherosclerosis using a 6-week semaglutide treatment regimen. Although human studies examining atherosclerosis remission with GLP-1RAs are limited, open-label observational studies of several hundred people with T2D treated with liraglutide (45) or exenatide once weekly (46) detected reduction in carotid intima media thickness, as assessed by carotid Doppler examination. In contrast, no change in carotid artery plaque volume or composition, as assessed by carotid MRI, was detected in 631 people with T2D randomized to receive placebo or exenatide once weekly for 18 months (47). Hence, it seems premature, based on the available evidence, to conclude that GLP-1RAs are uniformly capable of inducing atherosclerosis regression.

### Limitations of the work.

We have utilized mRNA transcript analysis to characterize elimination of *Glp1r* in various Tie2^+^ cell populations. We cannot be certain that these transcript levels always correspond with functional GLP-1R protein levels. Although we determined that a wide range of Tie2^+^ ECs and HL cells are not required for the antiatherogenic actions of semaglutide, our studies did not identify the precise GLP-1R^+^ cells required for GLP-1RAs to attenuate atherosclerosis. Similarly, although our data implicate one or more Tie2^+^ cell types as essential for the semaglutide-mediated reduction of hepatic inflammation and fibrosis, the precise identity of these GLP-1R^+^ cells remains to be determined. Selective elimination of the GLP-1R in γδ T cell lineages would be useful to further delineate the relevance of these cells to the immunomodulatory actions of GLP-1RAs. Moreover, although semaglutide did not induce regression of atherosclerosis, we were unable to detect any regression in mice switched back to a less atherogenic diet, limiting the available conclusions. Nevertheless, taken together, the current data set advances our understanding of the potential roles of Tie2^+^ GLP-1R^+^ cells as targets of GLP-1 action, being dispensable for the antiatherogenic actions of GLP-1, while critical for a subset of antiinflammatory actions in the liver.

## Methods

### Animal care, genotype, and treatment.

All mice were fed ad libitum a regular chow diet (no. 2018, 18% kcal from fat; Harlan Teklad) and water, unless otherwise specified, and housed under 12-hour-dark/light cycles. C57BL/6 WT mice were obtained from in-house breeding, and *Ldlr*^–/–^ mice (*Ldlr*^tm1Her^) were purchased from The Jackson Laboratory (stock 002207). Mice with *Glp1r* deletion under the regulation of the *Tie2* gene promoter (*Tek*) were generated by crossing Tg(Tek-Cre)1Ywa mice (Tie2-Cre) from The Jackson Laboratory (stock 008863) with *Glp1r*^fl/fl^ mice (48). Male mice with a single *Tie2*-Cre allele and homozygous for *Glp1r*^fl/fl^ were crossed with female mice homozygous for *Glp1r*^fl/fl^ to produce *Glp1r*^fl/fl^ (*Glp1r*^Tie2+/+^) and *Glp1r*^fl/fl^/Tie2-Cre (*Glp1r*^Tie2–/–^) littermates. *Rag2*/*Il2rg* double-knockout mice purchased from The Jackson Laboratory (stock 014593) were back crossed onto the BALB/c genetic background for 8+ generations, with WT littermates used as controls. *Rag2*/*Il2rg* double knockout mice were propagated as described previously (49).

HFHC feeding protocols used Envigo TD.88137, which has 42% calories from fat and 0.2% cholesterol. An established PCSK9-AAV method (38) of generating hypercholesterolemia and atherosclerosis was utilized in *Glp1r*^Tie2+/+^ and *Glp1*r^Tie2–/–^ mice: mPCSK9 (plasmid no. 58376; donated by Jacob Bentzon, Aarhus University, Aarhus, Denmark; ref. 50) was obtained from Addgene and produced with an AAV8 vector by Penn Vector Labs (full description: AAV8.ApoEHCR-hAAT.D377Y-mPCSK9.bGH). 3 × 10^11^ genomes were delivered in a single tail vein injection.

For studies of atherosclerosis and the actions of liraglutide, male *Ldl*r^–/–^ mice, starting at 8–9 weeks of age, were fed HFHC diet or regular chow and received daily s.c. injection of liraglutide or an equal volume of vehicle in a volume of 4 ml/kg for 18 weeks. Liraglutide dosing was started at 50 μg/kg/d for week 1, increasing to 100 μg/kg/d for week 2, and then maintaining at 200 μg/kg/d for the remainder of the study.

Female *Glp1r*^Tie2+/+^ and *Glp1r*^Tie2–/–^ mice received an injection of PCSK9-AAV at 8–9 weeks of age and were started on HFHC diet after 1 week. Mice were treated with daily s.c. injection of semaglutide or an equal volume of vehicle in a volume of 4 ml/kg for 18 weeks. Semaglutide dosing was started at 2.5 μg/kg/d for week 1, followed by 5 μg/kg/d for week 2, and 10 μg/kg/d for the remainder of the study.

For a model of atherosclerosis regression, female WT mice received PCSK9-AAV at 8–9 weeks of age; they were started on the HFHC diet after 1 week, and this diet was continued for 16 weeks. Mice were then switched to chow diet for 1 week, followed by daily s.c. injection of 10 μg/kg/d semaglutide or an equal volume of vehicle in a volume of 4 ml/kg for 6 weeks. Body composition was measured using a nuclear magnetic resonance system (EchoMRI).

### Tissue collection.

Mice were euthanized by CO_2_ inhalation; frozen tissues were collected by snap freezing in liquid nitrogen. Blood was collected by cardiac puncture and mixed with 10% v/v TED (5000 KIU/ml Trasylol, 1.2 mg/ml EDTA, 0.1 nmol/l Diprotin A). Blood was spun at 12,000*g* for 5 minutes for plasma collection.

Whole aortas were fixed with 10% neutral buffered formalin overnight followed by staining of atherosclerotic lesions with Sudan IV (MilliporeSigma, S-8756). All perivascular adipose tissue was removed, and aortas were opened longitudinally and pinned flat for imaging. Images were taken with a Sony a5000 camera with 30 mm F3.5 Macro lens. Aorta images were analyzed with ImageJ (NIH) for relative area of Sudan IV^+^ staining to total area. For liver histology, tissue was fixed in 10% neutral buffered formalin for 24 hours and transferred to 70% ethanol before paraffin embedding and Picrosirius red staining. Slides were scanned with an Olympus VS-120 slide scanner. Images were analyzed in QuPath using automated threshold function. All aorta and liver histology analysis was performed in a blinded manner.

### Glucose, lipid, GLP-1, and cytokine measurements.

For oral glucose tolerance test, mice were fasted for 5 hours, with semaglutide or vehicle administered at the start of fasting. Oral glucose gavage was administered at 1.5 g/kg with a concentration of 0.15 g/mL glucose in water. Glucose measurements (Contour glucometer) from tail vein blood were taken at 0, 10, 20, 30, 60, 90, and 120 minutes after glucose gavage.

Blood collection for plasma lipid profiling was performed in mice injected with PBS or semaglutide, followed by a 5-hour fasting period. Tail vein blood was collected in K3 EDTA-coated capillary Microvette tubes (Sarstedt); fresh unfrozen plasma was separated by fast protein liquid chromatography and analyzed for cholesterol and triglycerides as previously described (51). Fraction numbers 5–9 were labeled as VLDL, 10–18 as LDL, and 19–25 as HDL. Liver tissue triglyceride levels were measured as previously described (52).

For total GLP-1 measurements in fasted and nutrient-stimulated states, PCSK9-AAV– treated, HFHC diet–fed mice (for 15 weeks) were fasted for 5 hours before collection of tail blood in the fasted state, 10 minutes after 2 g/kg glucose in 20%w/v water was given orally. One week later, studies were repeated with 100 μl olive oil given orally. Total GLP-1 was measured using the V-PLEX GLP-1 Total Kit (Mesoscale, K1503PD).

Blood plasma cytokines were measured in endpoint blood collected by cardiac puncture with the V-PLEX Proinflammatory Panel 1 Mouse Kit (Mesoscale, K15048D) and a 10-cytokine panel for IFN-γ, IL-1β, IL-2, IL-4, IL-5, IL-6, KC/GRO, IL-10, IL-12p70, and TNF-α; IFN-γ, IL-1β, IL-2, and IL-4 were largely below the limit of detection and were not plotted. IL-17A detection was performed by ELISA (Biolegend, 432504).

### Aorta and liver cytometry and liver cell culture.

Freshly isolated whole aortas were cleaned of all perivascular adipose tissue and minced and mechanically disrupted with a GentleMACS dissociator (Miltenyi Biotec, 130-096-427), program 37C_m_TDK_2, using multitissue dissociation kit digestion buffer (catalog no. 130-110-201). Isolated cells were washed with FACS buffer (PBS with 2 mM EDTA, 25 mM HEPES, 2%v/v FBS). Fluorescence-conjugated antibodies were obtained from Biolegend: CD31 (clone 390); CD45 (clone 30-F11); TCRγδ (clone GL3); TCRβ (clone H57-597); CD4 (clone GK1.5); CD8α (clone 53-6.7); CD3 (clone 17A2); CXCR6 (clone SA051D1); and CD19 (6D5). Cells were sorted with a MoFlo Astrios Cell Sorter (Beckman) into FBS-coated tubes and pelleted and frozen for RNA analysis.

Livers were processed for FACS cytometry using enzymatic perfusion or only mechanical disruption. Enzymatic perfusion was performed as previously described for studies illustrated in Supplemental Figure 4, A and B, and ref. 52. Briefly, the liver was perfused with pronase and collagenase buffer via the inferior vena cava with the portal vein cut, and the superior vena cava was clamped. For specific collection of liver immune cells, a simplified mechanical disruption protocol was used in experiments depicted in Figure 5, A and B, and Supplemental Figure 4, C–F. Livers were first flushed by cutting the right atria and performing cardiac perfusion (35 mmHg) with PBS for 1 minute. Liver tissue was then minced and pressed through successive 200-micron and 70-micron filters in 50 mL cold RPMI+10% FBS media. The resulting cell suspension was pelleted (8 minutes, 400*g*) and resuspended in 37.5% Percoll (MilliporeSigma) in RPMI media (spun 20 minutes, 850*g*). NH cell pellets were resuspended in RBC lysis buffer (Biolegend) (10 minutes, room temperature). Washing and subsequent immunostaining were performed in FACS buffer. All cytometry gating was performed with FMO controls.

For ex vivo activation of liver NH cells, preparations as described above were resuspended in RPMI 1640 media (Gibco, reference 11875) supplemented with 10% FBS, 100 μM HEPES (Gibco, 15630106), NEAA (Gibco, 11140050) 100U/ml penicillin/streptomycin (Gibco, 15140122), 1 mM pyruvate, and 3.5 μl/L 2-mercaptoethanol and rested at 37°C for 30 minutes. Cells were then plated on culture dishes precoated with 5 μg/ml anti-CD3 (clone 17A2) or isotype control (clone RTK4530) with 1 μg/ml CD28 (clone 37.51) or PMA-ionomycin (Biolegend, 423301) and cultured overnight for 20 hours.

### RNA isolation and analysis.

Frozen tissues or cells were homogenized in Tri Reagent (MRC) using a TissueLyser II (Qiagen). Pancreas tissue was freshly homogenized in Tri Reagent before freezing. 500–1000 ng total RNA was treated with DNase1 (Thermo Fisher Scientific, EN0521), and cDNA was synthesized using random hexamers and SuperScript III (Thermo Fisher Scientific, 1808044), followed by qPCR for gene expression or regular PCR for amplification of the full-length *Glp1r* transcript. qPCR primers are shown in Supplemental Table 1. Gene expression was quantified for target genes compared with a reference gene using the 2^–ΔCT^ method, as specified in the figure legends. PCR amplification of a transcript encompassing the majority of the *Glp1r*-coding region was performed with 5′-AGAGACGGTGCAGAAATGGA-3′ forward primer and 5′-CTGTGGTCCTTGCTTCTGG-3′ reverse primer. After gel electrophoresis and transfer to a nylon membrane, blots were hybridized with a ^32^P-labeled (5′-GGATGGGCTCCTCTCCTAAT-3′) internal GLP-1R oligonucleotide probe.

### Statistics.

Graphing of results and statistical analyses were performed using GraphPad Prism 9. Biological replicates are shown as individual data points, with a bar graph representing the mean and error bars showing SD. Two-tailed Student’s *t* test was performed for comparison between 2 groups. Experiments involving variables for both genotype and treatment were analyzed by 2-way ANOVA and multiple comparisons for treatment (Bonferroni’s multiple-comparison test). Significance determined by multiple comparison is shown in graph annotations, with 2-way ANOVA results only shown if no significant differences were found with multiple comparison. *P* ≤ 0.05 was considered statistically significant.

### Study approval.

Mouse studies were performed in accordance with protocols approved by the Sinai Health System and The Centre for Phenogenomics.

## Author contributions

BAM, CKW, and KDK designed and executed the experiments and reviewed and edited the manuscript. DJD designed the experiments, and both BAM and DJD wrote the manuscript. RJS reviewed and edited the manuscript.

## Supplementary Material

Supplemental data

## Version 1 10/21/2021

In-Press Preview

## Version 2 11/22/2021

Electronic publication
