# Supplementary material for: Differential importance of endothelial and hematopoietic cell GLP-1Rs for cardiometabolic versus hepatic actions of semaglutide
Source: JCI Insight. 2021 Nov 22;6(22):e153732. doi: 10.1172/jci.insight.153732 (PMC8663785; doi:10.1172/jci.insight.153732)
Supplement: Supplemental data [file jciinsight-6-153732-s118.pdf]

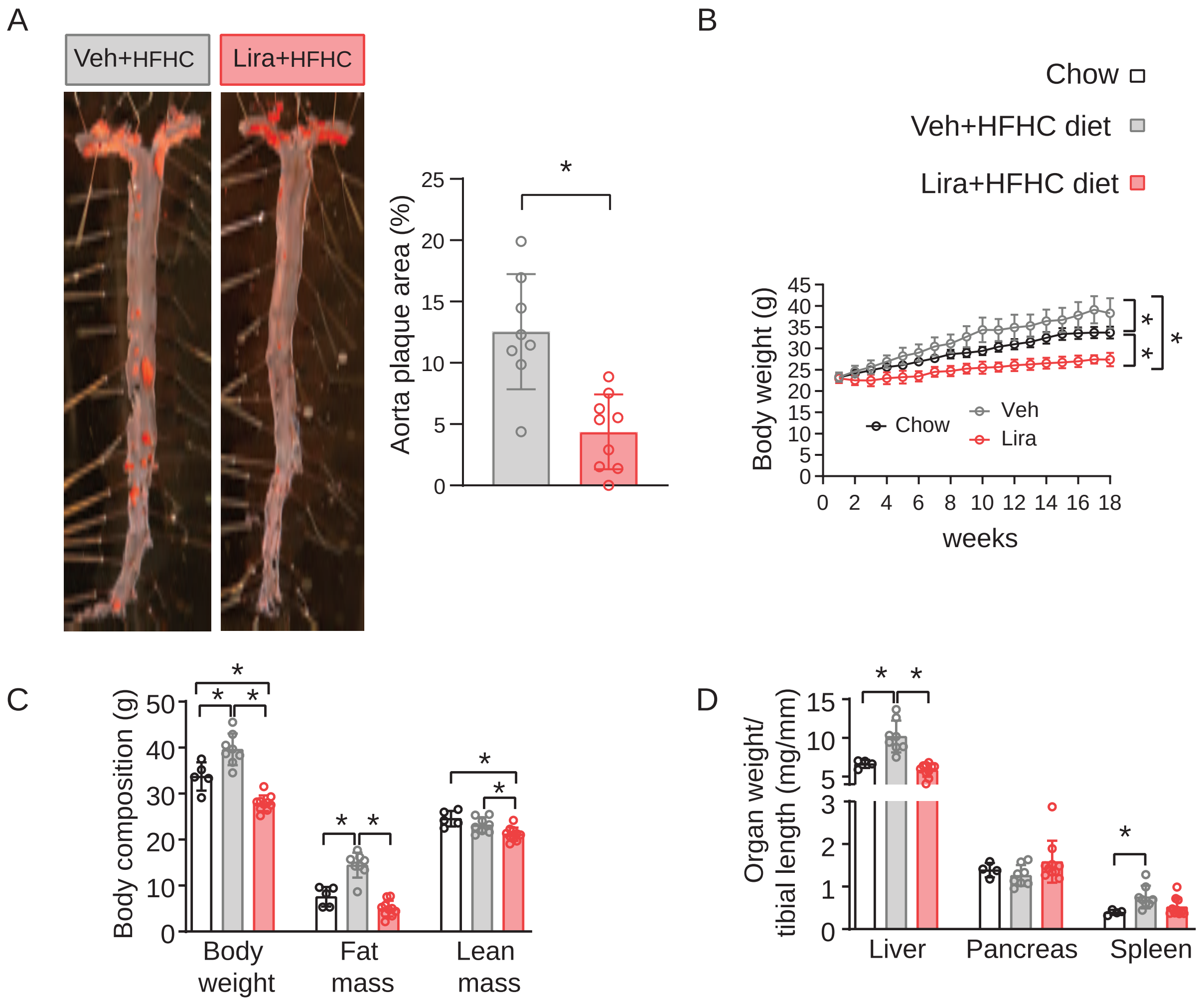

Supplemental Figure 1: Liraglutide reduces atherosclerosis and body weight in *Ldlr*$^{-/-}$ mice. *Ldlr*$^{-/-}$ mice were fed a HFHC diet for 18 weeks with daily S.C. treatment of either liraglutide (Lira; 200µg/kg) or equal volume vehicle (Veh) (n=8-10). Untreated, chow-fed mice were included for comparison. Whole aortas stained with Sudan IV for atherosclerotic plaques; representative images and quantification (A). Chow-fed mice did not develop visible aortic plaques (not shown). Weekly body weights (B). End point body weight and body composition (C), as well as end organ weight for liver, pancreas and spleen relative to tibial length were measured (D). Data presented as mean +/-SD with individual data points shown. *P<0.05 one-way ANOVA.

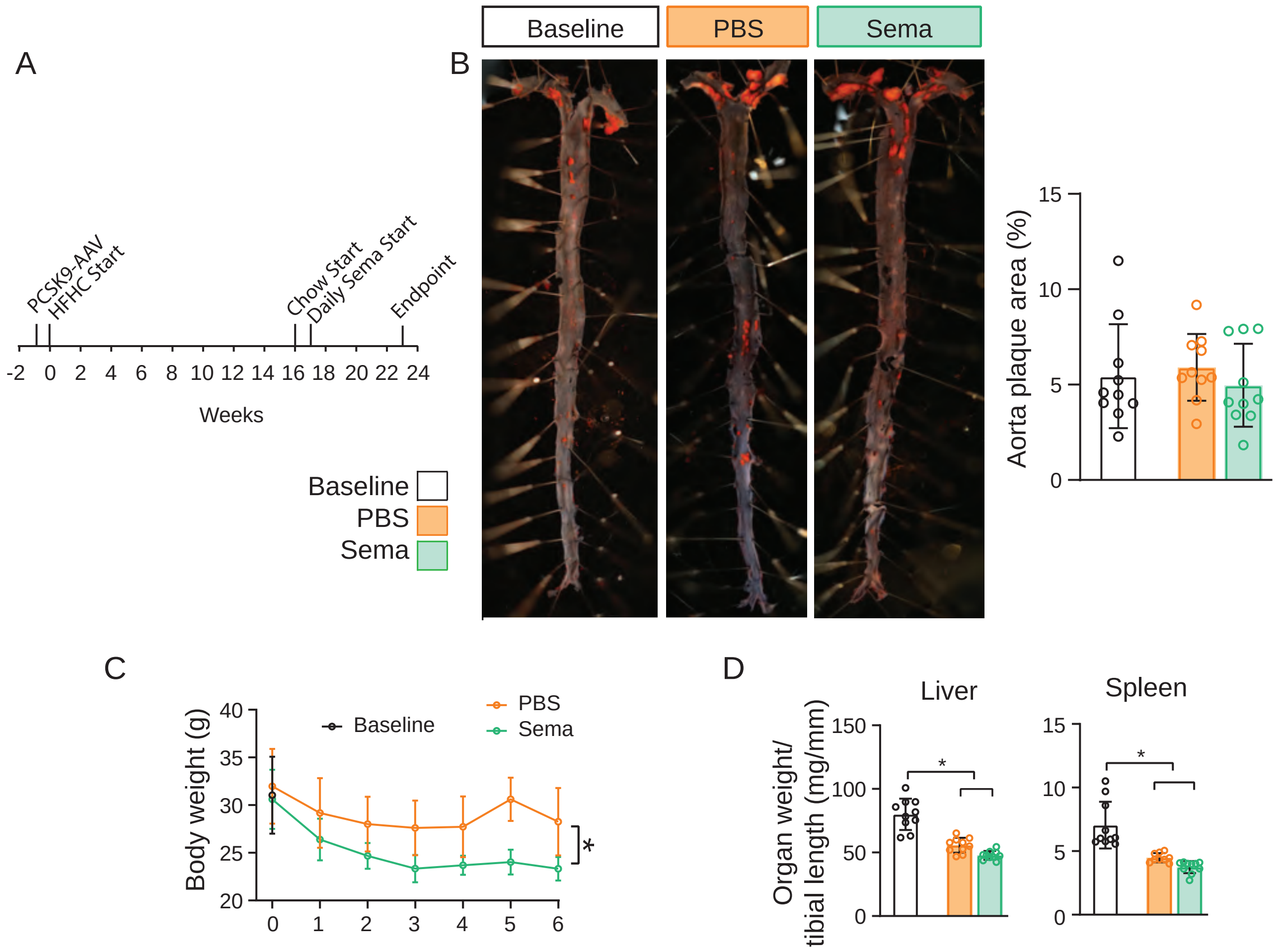

Supplemental Figure 2: Daily semaglutide treatment does not promote regression of atherosclerosis. Mice were given PCSK9-AAV and fed a HFHC diet for 16 weeks, before being switched to a chow diet for one week. A subset of mice was randomized for tissue collection at this point (baseline) or after 6 weeks continuing on chow diet with daily treatment of Semaglutide (Sema; 10µg/kg/day) or equal volume vehicle (Veh) (A). Whole aorta mounted *en face* stained with Sudan IV for detection of atherosclerosis plaques; representative images and quantification (B). Weekly body weight (C) and end point liver and spleen weight/tibial length (D). Data presented as mean +/-SD with individual data points shown. *P<0.05 one-way ANOVA.

A

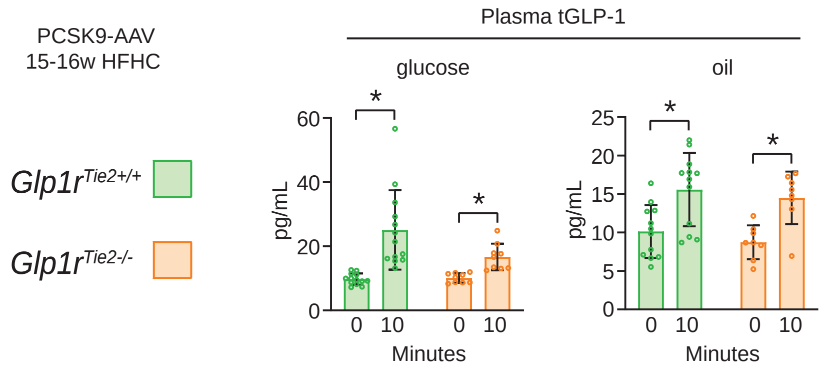

Supplemental Figure 3: *Glp1r*$^{\mathrm{Tie2-/-}}$ mice do not exhibit dysregulated control of plasma GLP-1 levels. *Glp1r*$^{\mathrm{Tie2+/+}}$ and *Glp1r*$^{\mathrm{Tie2-/-}}$ mice were given a single PCSK9-AAV injection, followed by HFHC diet feeding for 15-16 weeks. Blood was collected after 5hrs fasting (0) and 10min after mice received either oral glucose or olive oil (oil) for measurement of total Glp-1 (tGLP-1). (n=8-12). Data presented as mean +/-SD with individual data points shown. *P<0.05 for nutrient effect in a two-way ANOVA multiple comparison test.

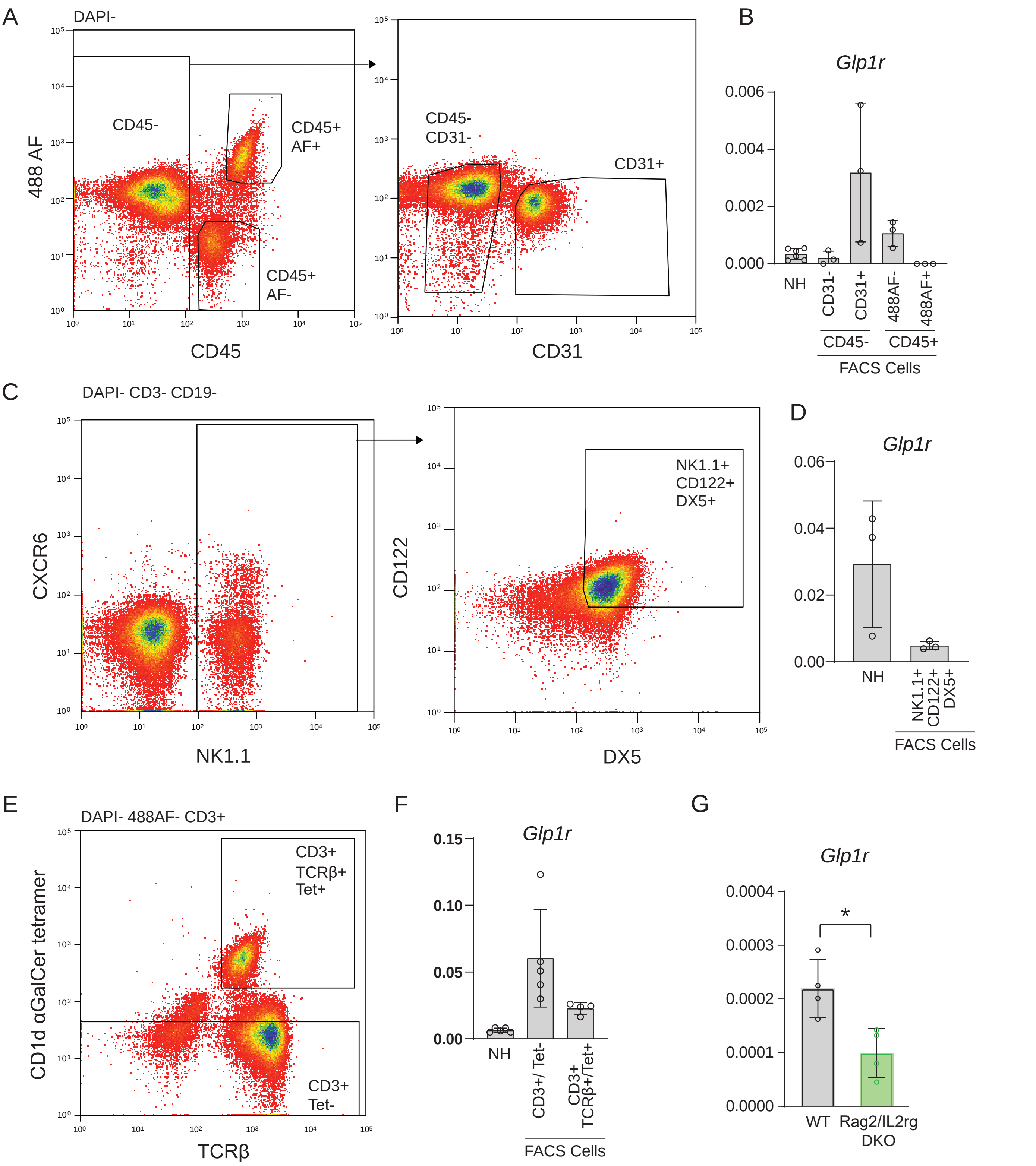

Supplemental Figure 4: Liver cell immune populations are enriched for *Glp1r* expression.

Cells from enzymatically digested perfused livers were subjected to FACS cytometry to collect major non-hepatocyte (NH) cell types. Fractions included CD45- non immune cells with CD31+ endothelial cells and remaining CD31- cells; CD45+ cells were divided into 488-526/52 channel auto fluorescent (AF)+ Kupffer cells and remaining CD45+ AF- cells; sorting gates (A) and RNA quantification for *Glp1r* expression relative to *Ppia* (B). Mechanically disrupted liver NH cells were sorted for NK cell markers: DAPI-, CD3-, CD19- and NK1.1+, CD122+, DX5+ (C); *Glp1r* expression relative to *Tbp* (D). Mechanically disrupted liver NH cells were sorted for NKT cells: CD3+, TCRβ+, CD1d αGalCer tetramer (Tet)+; CD3+, Tet- were collected for comparison (E); Glp1r expression relative to *Tbp* (F). Whole liver expression of *Glp1r*, relative to *Ppia*, compared between WT and *Rag2*$^{-/-}$/*Il2rg*$^{-/-}$ double knockout (DKO) mice (G) (n=3-6)). Data presented as mean +/-SD with individual data points shown. *P<0.05 Student's t-test for effect of WT vs DKO genotype.

Abbreviation	Full name	Catalogue number (Thermo)
ABCG1	ATP-binding cassette, sub-family G (WHITE), member 1	Mm00437390_m1
Adgre1	Adhesion G protein-coupled receptor E1 (F4/80)	mm00802529_m1
Ccl2	Chemokine (C-C motif) ligand 2 (MCP1)	Mm00441242_m1
Cd3g	CD3 antigen, gamma polypeptide	Mm00438095_m1
Col1a1	Collagen, type I, alpha 1	Mm00801666_g1
CRP	C-reactive protein, pentraxin-related	Mm00432680_g1
CXCR2	Chemokine (C-X-C motif) receptor 2	Mm00438258_m1
Glp1r	Glucagon-like peptide 1 receptor exon 5-6	Mm00445292_m1
Glp2r	Glucagon-like peptide 2 receptor x5-6	Mm01329475_m1
Il2	Interleukin 2	Mm00434257_m1
Il17a	Interleukin 17-alpha	Mm00439618_m1
Itgae	Integrin alpha E, epithelial-associated x20-21	Mm00434443_m1
Ifng	Interferon gamma	Mm01168134_m1
Lipc	Lipase, hepatic	Mm01171487_m1
Ppia	Cyclophilin	Mm02342430_g1
Rpl32	ribosomal protein L32	Mm02528467_g1
Slc27a5	solute carrier family 27 (fatty acid transporter), member 5 (FATP5)	Mm00447768_m1
Tbp	TATA box binding protein	Mm00446973_m1
TGFb1	transforming growth factor, beta 1	Mm01178820_m1
Tnf	Tumor necrosis factor alpha	Mm00443258_m1

Supplemental table 1
